# Topics of Public Interest

**Published:** 1912-09

**Authors:** 


### TOPICS OF PUBLIC INTEREST.

---

Examination of Dentists for the U. S. Army.—Examinations for the appointment of Acting Dental Surgeons will be held at Fort Slocum, New York; Columbus Barracks, Ohio; Jefferson Barracks, Missouri; Fort Logan, Colorado; and Fort McDowell, California, on Monday, October 7, 1912. For full information, address promptly the "Surgeon General, U. S. Army, Washington, D. C."

Acting Dental Surgeons are employed under a three years' contract, at the rate of $150.00 per month. They are entitled to traveling allowances in obeying their first orders, in changing stations, and in returning to their homes at termination of service. They also have the privilege of purchasing certain supplies at the Army commissary. After three years' service, if found qualified, they are promoted to the grade of dental surgeon with the rank of first lieutenant, and receive thereafter the pay and allowances appertaining to that rank.

---

1st International Congress of *Comparative Pathology* to be held at the Faculty of Medicine of Paris, from 17th to 23d October, 1912.

Among the principal subjects, which will fill the orders of business of the seatings, the following have already their reporters:

Tuberculosis: Prof. Calmette, Prof. Vallee, M. Chaussee; Human and Aviary Diphtheria: Prof. Arloing, Prof. Rappin; Cancer: Prof. Menetrier and Dr. Clunet, Prof. Borrel; Small-Pox and Vaccination: Director Chaumier, MM. Carriere and Tomarkin, (of Genova); Parasites Peculiar Both to Men and Animals: M. Weinberg, Prof. Deve, Prof. Bodin, M. Ch. Morot, Prof. Perroncito; Nervous Affections: Dr. Marchand and Prof. Petit; Hydrophobia: Dr. Delaunay, Dr. Remlinger, Prof. Babes; Comparative Study of Cirrhoses: Dr. M. Garnier, Dr. Ravenna; Pathology of Inferior Animals: Prof. Perroncito; Milk, Prof. Porcher, Dr. Henri de Rothschild; International Organization of the Struggle Against Foot and Mouth Disease: Prof. Moussu; International Organization of the Struggle Against Melitococoy, M. Oh. Durois; Vegetable Pathology: M. Louis Blaringhem, Dr. O. Larcher, M. Louis Dop, (of Roma).

All correspondence should be addressed to General Secretary: 42 rue de Villejust, Paris.

---

National Tuberculosis Sunday, Oct. 27, 1912.—The *National Association for the Study and Prevention of Tuberculosis* announces this date as the third annual attempt at education of the people in the matter of tuberculosis, from the pulpit. In 1911, out of some 200,000 churches in the United States, over 50,000 held special services and it is hoped that still greater interest will be shown this year. This movement has our most hearty support, though we cannot refrain from suggesting that "every minister in the United States should give this subject"

a great deal more than "some attention during the week preceding or following April 28." (The date originally planned.) It occurs to us that 50,000 men, however worthy and enthusiastic, but trained along entirely different lines from those of physicians and sanitarians, include a good many who would fail to make the most of the opportunities for presenting this subject and who might, by creating alarm among nervous persons, or by failing to grasp the practical etiologic and prophylactic principles involved, do a great deal of harm. We take the liberty to suggest that the address on tuberculosis be made by some physician chosen from the congregation.

A curious fact is noted in the circular issued by the Association. While the general tubercular mortality for the registration area of the U. S. 1.6 per mille, it was 2.24 per mille for 312 church members. Why should good people suffer more from tuberculosis than the wicked? Is it because of the increased labor and confinement incident to religious services, as compared with the hours of rest and open air sport enjoyed by the worldly? Or because the religious are more often engaged in the care of the unfortunate? Or because serious illness tends to awaken a sense of religious duty?

---

Infantile Paralysis in Buffalo.—Dr. Wade H. Frost of the U. S. Marine Hospital and Public Health Service has been investigating the present épidemic. State Health Commissioner E. H. Porter has called on the profession to co-operate in stamping out the disease by prompt diagnosis and reports.

That there has been considerable acute infantile paralysis in Buffalo and surrounding towns—Silver Creek, North Tonawanda, etc.—is true. That the State and National Health authorities are justified in sending representatives to study the disease, both from the standpoint of epidemiology, of clinical medicine and of bacteriology, is admitted. But that there is anything approaching a serious epidemic as might be concluded from some of the press reports, must be denied. The incidence of the disease, from reports alone, has been much higher proportionately to the population, in many cities and, as in every other instance in which there is a chance for sensationalism, the reports are in many instances based on erroneous diagnosis—one reputed case, for instance proving to be a slight traumatism from a fall. We are somtimes inclined to believe that women are not the only ones who are anxious to be in fashion. Years ago, when amœbic dysentery was in vogue, we made a number of observations for a clinician who was most anxious to establish a diagnosis. We could find nothing but leucocytes but a young friend very promptly found amoebae. At the same time, another physician lately promoted from the kitchen, put on record seven cases of amoebic dysentery from his own private practice. Then again, we can remember the time when malarial parasites were frequently found in the blood of Buffalo patients. Afterward, it was pretty conclusively demonstrated that there were no anopheles in the city. At any rate, either the amoebic dysentery and the malaria have died out or the interest in reporting them has. We hope the same will prove true of epidemic poliomyelitis and we are rather inclined to think that the genuine cases will ultimately be found not to represent a specific disease but to be due to the intracellularis, pneumococcus, or some other germ already well known in other sites. This is, however, a mere surmise and the subject deserves careful consideration while it is better to err on the side of too great apprehension.

---

Leper Removed From Buffalo. The leper discovered in Buffalo last month was, after consultation with various health authorities, spirited away from the city. The Common Council made some objection to paying transportation charges without definite information as to the place to which he was taken but it was thought best to keep the matter a secret. We hazard the guess that some university has brought influence to bear so as to keep the case under observation.

---

Hornell Water Pure. The state chemist has found the water supply free from dangerous organic and organized matter, though disagreeable in odor from the presence of algæ. The same algæ infest the Warsaw water.

---

Corning Free From Typhoid. As announced last month, the Corning water supply is now free from infection and the epidemic is past, while preparations are being made for an abundant supply of water from local deep wells, properly protected. One case of typhoid is being treated in the Buffalo Homoeopathic Hospital, contracted by a nurse from Buffalo in Corning.

---

Niagara River to Be Kept Free From Gross Contamination. The British Government has approved of the plan for an International Joint Waterways Commission, to regulate the disposal of sewage flowing at present into Niagara River. Buffalo is probably the chief source of danger though most of the typhoid in Buffalo is contracted outside the city. Many small

Canadian towns sewering into streams tributary to Niagara River or Lake Erie, also add to the danger. We are inclined to think that the compulsory diversion of Buffalo sewage from the river can be made a source of profit to the city. Sewage, handled on a large scale, is a valuable commodity. It would seem that, somewhere north of the city, a tract of cheap land could be bought, where sewage could be delivered mainly if not entirely by gravity, and that the sludge could be prepared for fertilization of slow growing crops—not vegetables and fruits—or that, following the plan of some European cities, the "drizzle fields" as they are so well termed in Germany could be themselves used as municipal farms. In this connection, we may mention that much of the garbage of Omaha is used to feed swine near the city, instead of being destroyed or "rendered."

---

28TH CHILD BORN.—A negress of Niagara Falls gave birth Aug. 7, to her 28th child. She has been married twice, is now 44 and was first married at 16. She has had triplets twice, twins three times.

---

RUPTURE OF BOWEL FROM AIR INTRODUCED PER ANUM.—Buffalo has recently had a second case of this sort, from the fooling of workmen with a compressed air hose. Death resulted in each case. A similar case was reported from Chicago several years ago and also by E. W. Andrews of Chicago in the *Jour. of Surgery., Syn. & Obstet,* Jan., 1911, with operation and recovery, and the accident has occurred a few other times. We understand also the inflation of the bowel and of the stomach with air, as well as distention with water, were formerly approved methods of torture. We were privileged to attend the necropsy on the first Buffalo case and, following it, made some observations on the patulousness of the anus, as it was surprising that the bowel could be distended to a dangerous degree by a blast of air simply held against the outer clothing over the anus. By using a stream of water under moderate pressure it was found that, especially with the body immersed in warm water, the current was sufficient to pass the sphinicter very readily, even when the nozzle of the hose was not in contact with the anus.

---

RAT PLAGUE AT NEW ORLEANS.—July 27, after several hundred negative examinations, bacilli of buconic plague were discovered in a rat. While no immediate fear of a spread of infection is entertained, it is disconcerting to learn that even one possible source has been found at our ports.

Right of Surgeon to Extend Operation According to Indications.—Judge Garrison of the Supreme Court of New Jersey, decided on July 14, that a surgeon has the right to use his judgment in extending an operation to meet indications. It should be noted, however, that this opinion is not necessarily binding in New York and there are certain features of the case to be considered; notably that the Judge was himself formerly a physician and that the suit which led to the decision was for damages under the technical term of assault and battery, by a charity patient, also that the modification of the operation was considered necessary to save life and that the patient was in good health at the time of the suit.

---

Limitation of Insurance Against Malpractice Suits.—Our attention has been called to the fact that certain policies against malpractice suits provide that the company shall not be responsible for damages and expenses in case of counter-suit against a suit for professional compensation. It is stated that this renders the insurance worthless in the great majority of all cases. Examine your own policy.

---

Pure Food Referee Decisions Final. — Attorney-General Wickersham has decided that no appeal can be made from decisions made by the Board composed of the Secretaries of Labor and Agriculture, Commerce and the Treasury.

---

Opposition to Immigration.—The American Federation of Labor, as part of its campaign to unionize the iron and steel industry and raise wages, advises its members in writing to friends abroad to discourage immigration for the next few years.

---

Increased Cost of Living.—On account of the advance in price of meat, New York restaurants have made a general increase of about 16% on meat orders, sometimes increasing the charge for vegetables also.

---

Sterilization of Criminals and Lunatics.—Indiana, California, Connecticut, Utah, Oregon, Iowa, New Jersey and New York have passed laws providing for the sterilization of the hopelessly unfit. The state medical societies of Florida, Louisiana and N. Dakota (homoeopathic in the last) have petitioned the legislatures for similar laws. The Texas *Medical Journal* (independent) is agitating for a similar law. It may be of interest to know that somewhat analogous laws existed in America many years ago In a book of travel two hundred years old, it is stated that one of the Central American tribes—and presumably others—entertained quite up-to-date notions as to morality. Any man who seduced a virgin was tried, and if found guilty, a twig of a thorn-bearing plant was introduced into his urethra and given a twist. In a hot climate infection, retention of urine and death was likely to follow. If not, the culpit was apt to behave himself for the future.

---

Anniversary Return of Lesions.—There is a popular impression that many lesions tend to return more or less exactly a year later, without repetition of the cause. Dr. Howard Crucher of Roswell, New Mexico, reports the lesions of diamond-back rattler bite, with diamond shaped patches, returning year after year; also a similar return of coyote bite. (*Am. Med.,* Aug., 1912). We have heard the same story about poison ivy but, in a moderate experience in camp, have never noted an anniversary return. At the risk of seeming credulous, we would ask for reports pro or con, in regard to the general topic of anniversary recurrence. It it is not impossible that an intense sui-suggestion might give rise to such phenomena, analogous to the classic stigmata.

---

Aftermath of the Titanic Disaster.—There can be no objection to a proper inheritance tax but it is somewhat disagreeable to think that we made money out of the loss of life from the Titanic, to the amount of about 45 cents for every man, woman and child in New York State.

---

Chickens in Cities.—Buffalo is agitating the matter of legislating against the keeping of chickens and it is proposed to have an ordinance by which two neighbors can compel the removal of a rooster that disturbs them by crowing. At first thought, it seems that the harboring of domestic animals in a city ought to be forbidden by law but there are some arguments on the other side. Chickens do not readily and essentially contract and disseminate any human disease. Care of poultry is profitable even on a small scale. It is often a very efficient aid against disease to have a few fresh eggs a day and an occasional non-storage fowl. Keeping chickens is good training for boys and girls. With the high cost of foods, every egg and every chicken produced is a benefit, even to those who would not themselves keep poultry. There are a great many noises quite as objectionable as cackling and crowing and nervous persons who would be awakened too early by roosters, would very likely already be awake on account of horseless and muffler-less vehicles, whose ubiquity at all hours of the night impresses every night-worker. Roosters can be rendered practically noiseless by section of the very accesible vocal cords—an operation which is very simple and almost painless.

---

FEE SPLITTING TABOOED IN TEXAS.—The State Medical Association has made fee splitting " an offense punishable by reprimand, suspension or expulsion" of a member.

---

The *United States Civil Service Commission* announces examinations to be held on September 11, 1912, at various places. Male physicians are needed at nine posts in the Indian service, salaries $1,000-$1,200.

---

CANCER BACILLUS discovered by Dr. Gaston Odin of Paris, positively identified and successfully cultivated; vaccine hoped for. This is the gist of press dispatches of Aug. 14, 1912. Having enthused at intervals for some years over the discovery of cancer germs, all we have to say is "may be."

---

LOSS IN WAGES BY SICKNESS. The N. Y. Dept. of Labor estimates that $366,000,000 was lost by 13,400,000 wage earners in the U. S. last year. Allowing for the fact that many included are probably permanently and hopelessly disabled, the per capita loss of less than $30 does not seem improbable but it is much higher than it should be. Many wage earners "lay off" with astonishing facility on account of what seem to more stoic persons, to be very trivial ailments. And, a good deal of reported sickness is simply drunkenness, or an excuse for idleness without any ground in fact. The same applies to persons not classed as wage earners. It is only fair to consider, too, that a headache, bruised finger, crick in the back, etc., which a professional or business man can afford to ignore, actually disable one engaged in mental detail work or manual labor. Moreover, the former can favor himself by postponing part of his work for a day or two, by telephoning, or by relegating details to assistants whereas the latter must actually lose time.

The N. Y. Association for Labor Legislation (Paul Kennaday, Secretary, 131 E. 23d St., N. Y. is making a systematic effort to learn what part of this great loss is due to preventable indisposition on account of bad ventilation, poisonous vapors, mental and physical overstrain, machinery traumatisms, etc.; and also to instruct and, if necessary, compel employers to remedy such defects. A booklet on Industrial Diseases will be mailed to every physician in the state and we bespeak the assistance of every one, in this philanthropy.

PYGOMELIA IN MAN, Dr. A. Baudrimont, *Jour. de Med. de Bordeaux,* June 16 and 23, 1912. This is an interesting review of literature beginning with an illustration of a three-legged duck and of a four-legged cow, or rather hermaphrodite bovine, since there was a common anus, a vulva of the autosite and a testicle and penis of the parasite. The case of Frank Lentini, a well built man with three legs is described and illustrated. A full report is made of the case of Miss S., for the first time, though including notes previously made by Pean and others. She was born in 1860 and died in 1880 from septicaemia originating in ulcers on the pygomelic leg. Illustrations are given showing the condition at the age of ten and shortly before death. The necropsy was secured with considerable difficulty owing to the desire of the "barnum" to exhibit the cadaver. In brief, the principal abnormalities consisted of a third leg, the stump of another, two vulvas, two functionating urethras, two bladders, two kidneys but one atrophic, two uteri, a pelvic tumor containing none of the ordinary features of a dermoid cyst but apparently representing small intestine, an external, pelvic, supernumerary mamma. We recommend a close study of this deport by those interested in teratology and will hold the originals for consultation for a short time, after which they may be consulted at the Grosvenor Library.

